# Disabling Pruritus in a Patient With Cervical Stenosis

**DOI:** 10.5435/JAAOSGlobal-D-19-00178

**Published:** 2020-03-09

**Authors:** Stephan N. Salzmann, Ichiro Okano, Jennifer Shue, Alexander P. Hughes

**Affiliations:** From the Department of Orthopaedic Surgery, Hospital for Special Surgery, New York, NY.

## Abstract

Brachioradial pruritus is a rare condition characterized by chronic localized itching of the dorsolateral upper extremities. Although the exact pathophysiology is still unknown, cervical nerve compression is thought to be a cause. We present the case of a 56-year-old man with a 6-year history of disabling chronic bilateral upper extremity pruritus and pain as well as concurrent neck pain. The patient presented to our office after multiple inconclusive diagnostic evaluations (dermatology, rheumatology, neurology, and psychiatry) and unsatisfactory multimodal conservative treatment attempts. His symptoms markedly impeded his ability to get restful sleep. Imaging of the cervical spine revealed multilevel cervical spondylosis, spinal stenosis with cord compression, and multilevel foraminal stenosis. The patient underwent successful multilevel anterior cervical decompression and fusion and was instantly symptom-free. The present case highlights that patients complaining of itching of the dorsolateral forearms of seemingly unknown etiology should undergo a workup of the cervical spine. If conservative treatment fails, surgical decompression may be considered in select patients.

Brachioradial pruritus (BRP) is a form of chronic pruritus that affects the dorsolateral aspect of the arms and forearms.^1^ Although the pathophysiology of BRP is a matter of debate, nerve compression in the cervical spine is thought to be a cause.^2^ The constant itching can have a serious debilitating effect on patients' quality of life. Oftentimes, patients undergo exhaustive diagnostic workup and conservative treatments with varying degrees of success.^3^ To the authors' knowledge, no reports exist in the orthopaedic literature on patients with severe BRP undergoing surgical treatment. We present a patient with cervical nerve root and cord compression causing disabling bilateral BRP who underwent multilevel anterior cervical diskectomy and fusion (ACDF) surgery and was instantly symptom-free.

## Case Report

**Figure 1:**
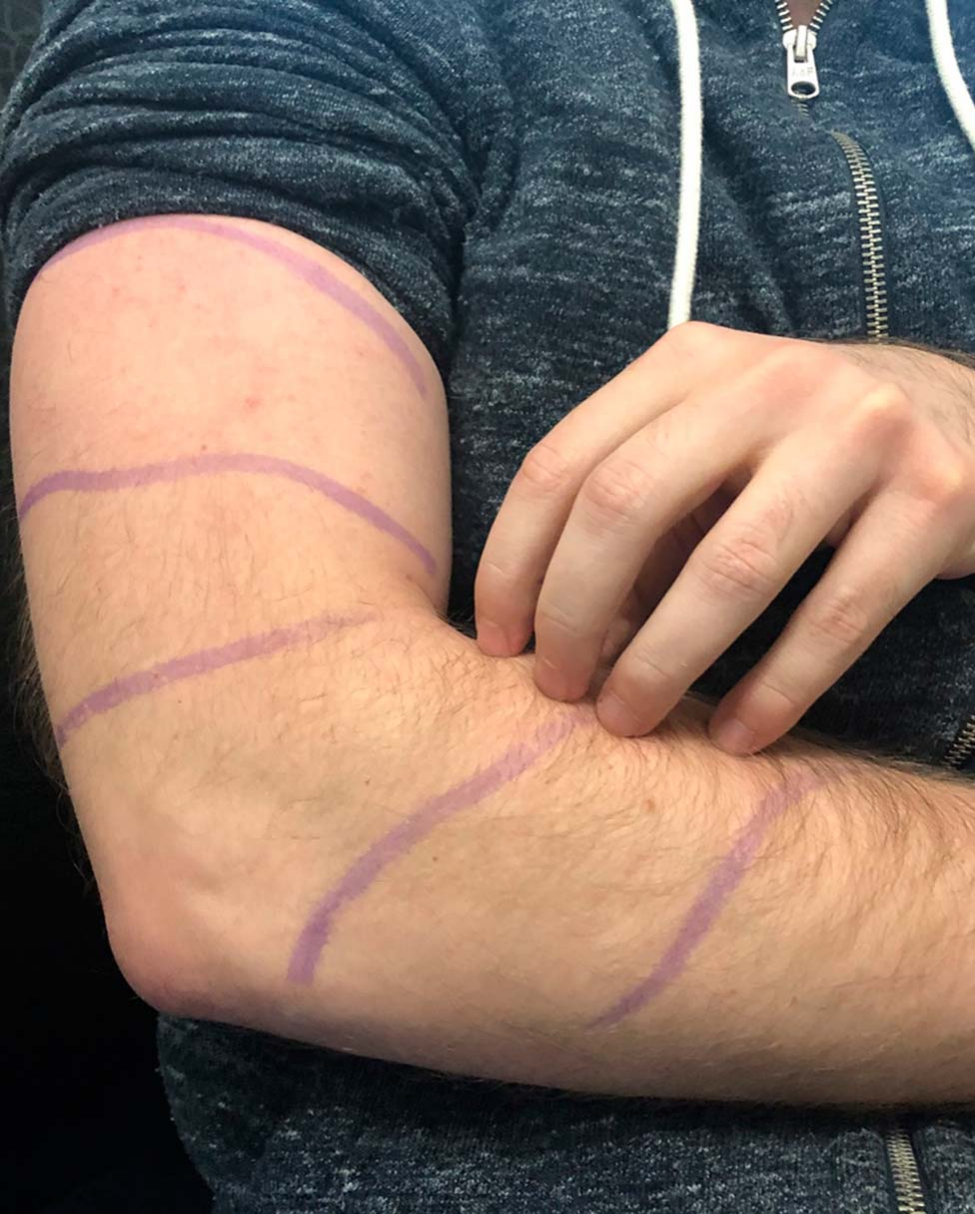
Photograph showing the typical region of pruritus demarcated in pen.

**Figure 2:**
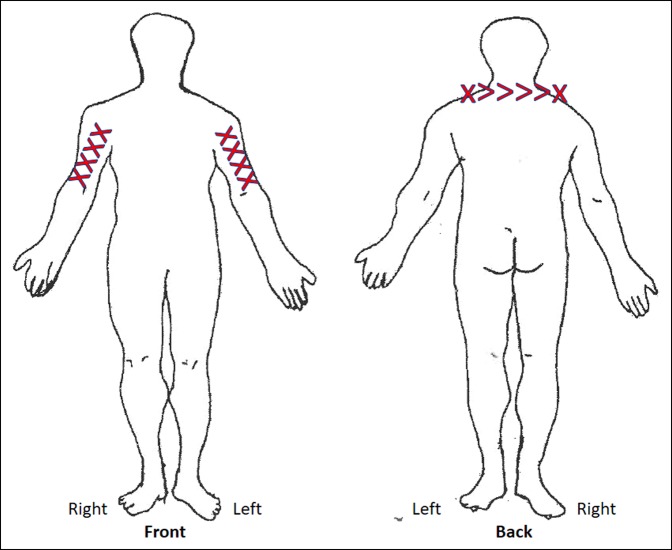
Illustration showing the areas affected by burning (indicated by x) and aching (indicated by >) as marked by the patient at his preoperative office visit.

A 56-year-old male nurse practitioner presented to our office with disabling pruritus affecting his bilateral upper extremities (Figure 1). The pruritus developed over the past 6 years and was concurrent with increasing neck pain (Figure 2). The patient underwent extensive evaluation including dermatology, rheumatology, neurology, and psychiatry. He had no evidence of either dermatologic disease or peripheral neuropathy, and his regular medications did not cause the pruritus. He had extensive multimodal conservative therapies including numerous epidural steroid injections that offered only transient relief.

The patient reported constant bilateral neck pain and intermittent bilateral upper extremity pain, burning, and pruritus, which extended into the bicep in a C6 dermatomal distribution. The symptoms worsened with reaching and lateral rotation of the neck, and decreased with ice application. The patient noted limited range of motion with right and left lateral rotation. He also reported of pins and needles and numbness in his bilateral upper extremities, which was positional. He noted no difficulty with dexterity, fine motor movements, or balance. His symptoms markedly impeded his ability to get restful sleep.

On physical examination, the area of itching skin was grossly normal. Neurologic examination was notable for a positive Spurling sign bilaterally. He had 5/5 strength in the major myotomes of the upper extremities and a 2+ bicep and triceps reflex response on a symmetric fashion. He denied decreased sensation in the distal dermatomes.

**Figure 3:**
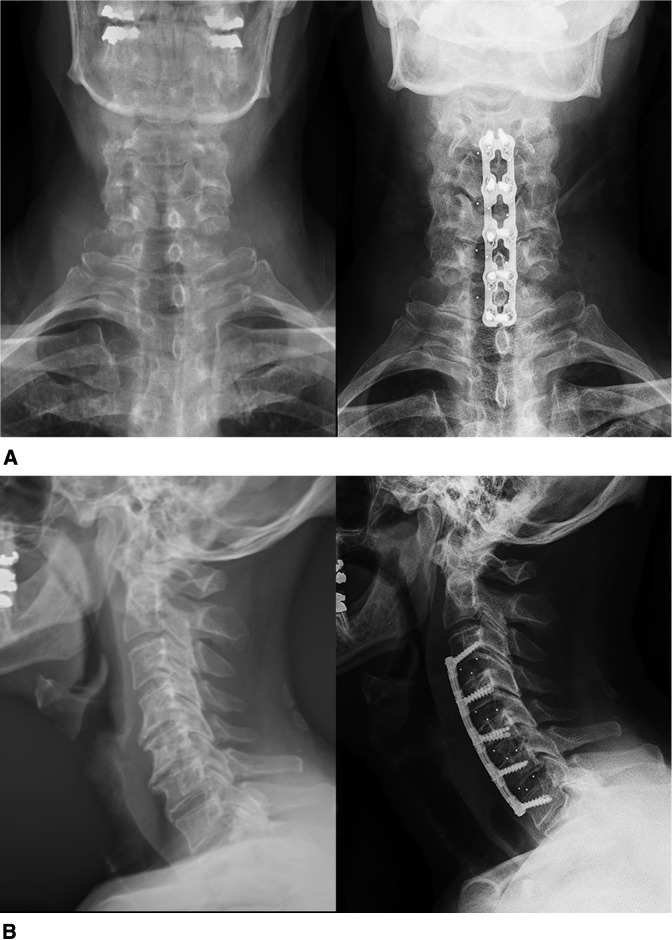
Radiographs of the cervical spine showing (**A**) preoperative (left) and postoperative (right) AP views and (**B**) preoperative (left) and postoperative (right) lateral view.

**Figure 4:**
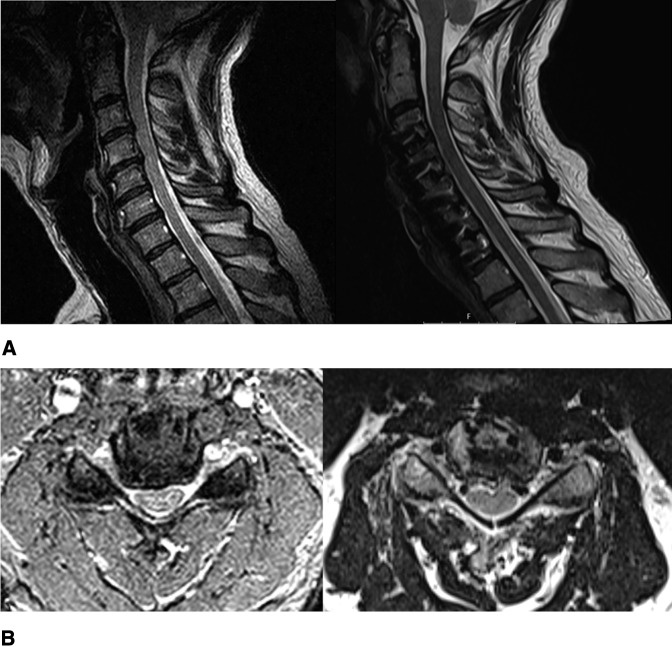
MRI scan of the cervical spine showing (**A**) sagittal view: (left) preoperative image demonstrating central spinal canal stenosis at C3-4 and C4-5 without obvious cord signal change, (right) postoperative. **B**, Axial view of the C4-5 level: (left) preoperative image demonstrating right-side dominant foraminal stenosis and central spinal canal stenosis with cord compression, (right) postoperative.

Spinal radiographs revealed multilevel cervical spondylosis (Figure 3). MRI showed notable spinal stenosis with early cord flattening at C3-4 and C4-5. In addition, multilevel foraminal stenosis from C3 to C7 was present (Figure 4).

The patient's chronic axial mechanical neck pain and bilateral upper extremity radiculopathy correlated with the degenerative and stenotic segments from C3 through C7. The pruritus was possibly caused by compression of the nerve roots and/or spinal cord at the affected levels. Given his multiyear history of multimodal conservative therapies and correlation between imaging and physical examination, it was thought that surgical intervention in the form of an ACDF from C3 to C7 would provide the patient permanent relief of his pain and pruritus. The surgical decision was made to address all levels with notable spinal and foraminal stenosis because they might have potentially contributed to the patient's symptoms. An anterior approach was selected based on the patient's radiographic features (including multilevel foraminal stenosis) and clinical symptoms in addition to the treating surgeon's good experience and familiarity with multilevel ACDF. The planned procedure was done without complication.

After surgery, the patient's symptoms improved instantly. At his most recent visit four months postoperatively, the patient related 95% improvement in his preoperative symptoms without any notable pain and only occasional sensation of pruritus.

## Discussion

The exact pathophysiology of BRP is poorly understood; however, two main mechanisms have been described.^2,3^ Waisman,^4^ who first reported this condition, thought that exposure to ultraviolet light was the main cause. Hence, the condition was also termed “solar pruritus.” Several years later, Heyl^5^ suggested that although sun exposure might contribute to the symptoms, the main etiologic factor is nerve injury because of cervical spine degenerative changes or nerve compression by other structures.

This hypothesis is supported by several reports of BRP in patients with cervical spondylosis, disk herniations, spinal tumors, and cervical ribs.^1,2,6-8^ In addition, as in the case presented here, decompression of neural structures may lead to instant symptom relief.^2^

The clinical diagnosis of BRP is based on its unique signs and symptoms.^2^ As in the presented case, symptoms usually develop bilaterally,^1,3^ are classically localized in a C5/C6 dermatomal distribution,^1^ and are markedly relieved by applying ice to the affected areas. This relief by ice is called the “ice pack sign” and is almost pathognomonic.^9^

Because BRP is a rare condition, there is a paucity of high-quality studies to guide evidence-based treatment.^2^ Conservative symptomatic treatments with topical steroids, antihistamines, NSAIDs, capsaicin, and gabapentin have shown varying degrees of success.^2,3,10^ In the presented case, cause-based treatment was done because of the clear evidence of compression of neural structures.

BRP is a unique neuropathic condition, which spine surgeons should be aware of. The case highlights that patients complaining of itching of the dorsolateral forearms of seemingly unknown etiology should undergo workup of the cervical spine. If conservative treatment fails, surgical decompression may be considered in select patients.
